# Case Report: Liver Transplantation in Homozygous Familial Hypercholesterolemia (HoFH)—Long-Term Follow-Up of a Patient and Literature Review

**DOI:** 10.3389/fped.2020.567895

**Published:** 2020-10-09

**Authors:** Matej Mlinaric, Nevenka Bratanic, Vlasta Dragos, Ajda Skarlovnik, Matija Cevc, Tadej Battelino, Urh Groselj

**Affiliations:** ^1^Department of Pediatric Endocrinology, Diabetes and Metabolic Diseases, University Children's Hospital, University Medical Centre Ljubljana, Ljubljana, Slovenia; ^2^Department of Dermatovenereology, University Medical Centre Ljubljana, Ljubljana, Slovenia; ^3^Department of Vascular Diseases, University Medical Centre Ljubljana, Ljubljana, Slovenia; ^4^Faculty of Medicine, University of Ljubljana, Ljubljana, Slovenia

**Keywords:** homozygous familial hypercholesterolemia, HoFH, fh, LDL-apheresis, liver transplantation, Slovenia, review

## Abstract

Homozygous familial hypercholesterolemia (HoFH) is a rare inherited metabolic disorder, frequently leading to an early cardiovascular death if not adequately treated. Since standard medications usually fail to reduce LDL-cholesterol (LDL-C) levels satisfactorily, LDL-apheresis is a mainstay of managing HoFH patients but, at the same time, very burdensome and suboptimally effective. Liver transplantation (LT) has been previously shown to be a promising alternative. We report on a 14 year-long follow-up after LT in a HoFH patient. At the age of 4, the patient was referred to our institution because of the gradually increasing number of xanthomas on the knees, elbows, buttocks, and later the homozygous mutation c.1754T>C (p.Ile585Thr) on the LDL-receptor gene was confirmed. Despite subsequent intensive treatment with the combination of diet, statins, bile acid sequestrant, probucol, and LDL-apheresis, the patient developed valvular aortic stenosis and aortic regurgitation by 12 years. At 16 years, the patient successfully underwent deceased-donor orthotopic LT. Nine years post-LT, we found total regression of the cutaneous xanthomas and atherosclerotic plaques and with normal endothelial function. Fourteen years post-LT, his clinical condition remained stable, but LDL-C levels have progressively risen. In addition, a systematic review of the literature and guidelines on the LT for HoFH patients was performed. Six of the 17 identified guidelines did not take LT as a treatment option in consideration at all. But still the majority of guidelines suggest LT as an exceptional therapeutic option or as the last resort option when all the other treatment options are inadequate or not tolerated. Most of the observed patients had some kind of cardiovascular disease before the LT. In 76% of LT, the cardiovascular burden did not progress after LT. According to our experience and in several other reported cases, the LDL-C levels are slowly increasing over time post LT. Most of the follow-up data were short termed; only a few case reports have followed patients for 10 or more years after LT. LT is a feasible therapeutic option for HoFH patients, reversing atherosclerotic changes uncontrollable by conservative therapy, thus importantly improving the HoFH patient's prognosis and quality of life.

## Introduction

Familial hypercholesterolemia (FH), the most common autosomal dominant condition, is due to the defective LDL-receptor leading to a decreased clearance of LDL-cholesterol (LDL-C) from plasma. In consequence, there is 2–3-fold elevation in the levels of total cholesterol (TC) and LDL-C after birth (1). Although the heterozygous FH (HeFH) is common (1/200–1/500), homozygous FH (HoFH) is a rare disease, affecting only four to six people in a million (2). Patients with HoFH can develop cutaneous and tendon xanthomas, arcus cornealis, and progressive generalized atherosclerosis in their early childhood. If untreated, patients with HoFH develop vascular lesions and cardiovascular disease (CVD) before the second decade of life and die before the end of the third decade of life (3, 4). Slovenia is, according to the available data, the only country with implemented nationwide universal screening for FH in preschool children, detecting both HeFH and HoFH patients (5–8).

HoFH is very difficult to manage. The medical treatment combines several cholesterol-lowering drugs used in other hypercholesterolemias. Initially, statins with ezetimibe are introduced and, in responsive patients, also PCSK9 inhibitors. Frequently, they do not result in satisfactory reductions in either TC or LDL-C levels, especially in moderate and severe HoFH patients with the highest CVD risk (9, 10). For over 30 years, LDL-apheresis is used, becoming a mainstay in the management of HoFH. It is currently considered the only safe and effective treatment for HoFH (11, 12). If LDL-apheresis is not successful, liver transplantation (LT) can be an alternative, also considering that LT is shown to be a successful treatment option in other metabolic liver diseases (13).

We aimed to report on a 14 year-long follow-up after LT in a HoFH patient at our center. In addition, a systematic review of the literature on LT for HoFH patients was performed.

## Methods

We collected all the available clinical information of a now 31 year-old male patient, who was followed at the Department of Endocrinology, Diabetes, and Metabolism of the University Children's Hospital Ljubljana, UMC Ljubljana, Slovenia from the age of 4. FH in the patient was detected incidentally by the dermatologist before implementing the Slovenian universal screening program (5–8). The medical records were collected and entered into the national FH registry database with informed consent from the patient. Genetic analysis was performed after obtaining informed consent as a part of a prospective study on clinical and genetic characteristics of FH patients approved by the National Medical Ethics Committee [the genetic analysis was explained previously in detail by Klančar et al. (6)].

For the systematic review, two approaches were applied. For the case reports on LT as a treatment for FH patients, the PubMed database was used. The following search terms were used: “liver transplantation” (AND) [“familial hypercholesterolemia” (OR) “homozygous hypercholesterolemia” (OR) “familial hyperlipidemia”]. We found 111 research articles. By reading all the abstracts and titles, we excluded 93 articles that did not meet the following conditions: (1) only articles in English and articles published after 1998 were used, but no limits were made on the country of research; (2) only articles that were fully accessible were included; (3) only the articles on humans and not on animals nor cell models were used; (4) only articles where the recipient of the liver transplant has had FH were used; and (5) only articles with follow-ups longer than 1 month were included. All the available articles that met the criteria were read in full-text form. In addition, other case reports were found through the articles' reference list. In the end, 23 articles were included. A systematic review of the clinical guidelines on LT was based on the search procedure used by Migliara et al. (14). In the end, we have found 17 guidelines.

## Case Description

**Figure 1:**
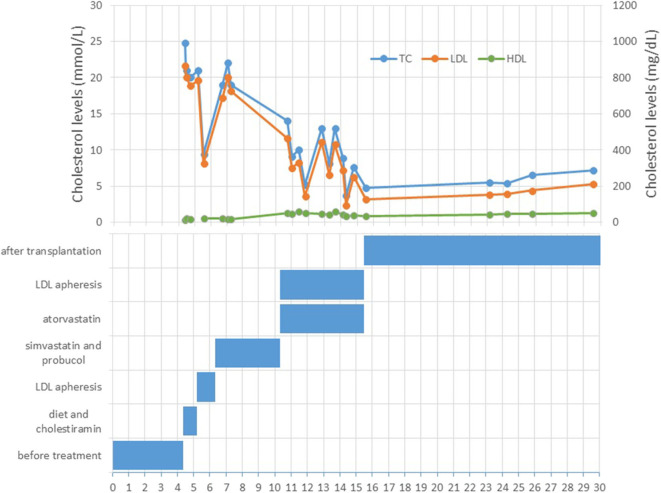
Overview of the cholesterol levels and therapy through the patient's lifetime.

A 4 year-old male patient was referred to our institution by a dermatologist because of the gradually increasing number of xanthomas on the knees, elbows, and buttocks that first appeared when he was 3 years old. He was born after an uneventful pregnancy and delivery as the second child of apparently non-consanguineous parents. The family history for premature CVD was negative. His early development was normal. Extremely high levels of TC (24.8 mmol/L; 928 mg/dl), LDL-C (21.6 mmol/L; 834 mg/dl), and triglycerides (TGC) (5.0 mmol/L; 443 mg/dl) and low levels of HDL-cholesterol (HDL-C) (0.3 mmol/L; 11.6 mg/dl) were measured at the first exam. Elevated levels of TC and LDL-C were also found in his parents, sister, and daughter (afterward) (Table 1). The clinical and biochemical picture suggested the diagnosis of HoFH, which was later confirmed genetically; a previously described homozygous mutation c.1754T>C (p.Ile585Thr) was found in the patient, while both his parents, his sister, and daughter were confirmed to be heterozygotes for the same mutation. Despite treatment with a highly restrictive diet, cholestyramine, statins, and LDL-apheresis (the exact timeline is described in Figure 1, Table 2), xanthomas extended progressively. At 7 years, increased IMT of the common carotid artery was measured with ultrasound. At 12 years, echocardiographically valvular aortic stenosis and aortic regurgitation were observed. Levels of LDL-C at the time of LDL-apheresis ranged from 2.4 to 11.6 mmol/L (92.8–449 mg/dl) in between treatments. Lp(a) levels ranged from 756 mg/L at the beginning to 135 mg/L after the last treatment. He refused to have apheresis for 3 years, but with psychological support, he continued.

**Table 1:** Plasma levels of total, LDL, HDL cholesterol, and Lp(a) in patient, parents, and sister at the same time point (in 2012 and PWV values in 2016).

	**Patient (23 years*^a^*)**	**Mother (42 years*^a^*)**	**Father (49 years*^a^*)**	**Sister(25 years*^a^*)**	**Daughter (3 years*^c^*)**
FH defect	Homozygous	Heterozygous	Heterozygous	Heterozygous	Heterozygous
Therapy	Liver transplantation	None	None	None	None
TC (mmol/L) TC (mg/dl)	5.5 (24.8c*^c^*) 213 (959c*^c^*)	8.2 317	11.0 425	10.4 402	6.9 267
LDL (mmol/L) LDL (mg/dl)	3.8 (21.6c*^c^*) 147 (835c*^c^*)	6.0 232	8.9 344	7.7 298	5.1 197
HDL (mmol/L) HDL (mg/dl)	(0.3*^c^*) 42.5 (11.6c*^c^*)	1.5 58	1.3 50	1.5 58	1.4 54
Tg (mmol/L) Tg (mg/dl)	1.4 (5*^c^*)	1.5 133	1.5 133	2.6 230	1.0 88
Lp(a) (mg/L)	111	<93.1	1260	687	323
cIMT (mm)	Left 0.6 Right 0.6	Left 1.4 Right 1.4	Left 0.7 Right 0.7	Left 0.5–0.6 Right 0.6	NS
Carotid stenosis	No	Yes	No	No	NS
PWV (m/s)*^b^*	4.6 ± 0.2	7.4 ± 0.7	8.9 ± 0.6	4.9 ± 0.2	NS

*In 2019 his daughter was referred to our center by cascade screening*.

a *Age in 2012*.

b *Measured in 2016*.

c *Measurements at the first referral*.

**Table 2:** Timeline of clinical event.

**Age**	**Description**
3	Xanthomas were found by the dermatologist
4	First examination at the tertiary clinic (total cholesterol: 2.8 mmol/L, 959 mg/dl) a diet and cholestyriamine were prescribed
5	Biweekly LDL-apheresis was started, xanthomas extended
6	The appearance of psychological problems, treatment with probucol and simvastatin, progression of xanthomas
7	Increased cIMT was first observed
9.5	Psychological support was introduced, started again with LDL-apheresis
12	A new systolic heart murmur was found, valvular aortic stenosis and aortic regurgitation were observed
16	Ortopic liver transplantation
16.5	Steroids after transplantation where gradually tapered, xanthomas had regressed, TC was in the normal range
17.5	TC was at the lowest value 4.4 mmol/L (170 mg/dl)
18	An episode of acute rejection developed, successfully treated with steroids. At the end of the treatment, TC was 5.1 mmol/L (197 mg/dl)
25	Cutaneous xanthomas completely resolved, complete regression of aortic stenosis
28	Pulse wave velocity was in the normal range (4.6 ± 0.2 m/s)
29	cIMT was 0.903 mm, TC was 7.2 mmol/L (278 mg/dL), statins were prescribed again

Due to progressive worsening conditions (valvular aortic stenosis and aortic regurgitation), the patient underwent deceased-donor orthotopic LT when he was 16 years old. There were no intraoperative or postoperative complications. The patient's immunosuppression regimen first consisted of tacrolimus, methylprednisolone, and ursodeoxycholic acid, but the steroid was gradually tapered. Six months after the LT, the patient's xanthomas had regressed, and the TC levels have fallen to 5.2 mmol/L (201 mg/dl). A year and a half after the LT, the TC level was at the lowest value [4.4 mmol/L (170 mg/dl)]. Two years after the LT, an episode of acute rejection developed, which was successfully treated with steroids, and the TC level was measured at 5.1 mmol/L (197 mg/dl).

Eight years after the LT, the patient who was then 24 years old was doing well. The patient had been on maintaining tacrolimus therapy. His cutaneous xanthomas completely resolved. An echocardiogram showed complete regression of aortic stenosis, and his IMT was in the normal range (0.6 mm on both common carotid arteries). His TC and LDL-C levels were 5.4 and 3.9 mmol/L, respectively (209 and 151 mg/dl), and Lp(a) levels were 111 mg/L. His LDL-C levels were lower than the LDL-C levels measured on the same occasion in his parents and sister (Table 1). Twelve years after the LT, the PWV velocity was measured with ultrasound, and the values were in the normal range (4.6 ± 0.2 m/s) The RHI was 2.46.

Afterward, the patient was not responding to the invitations from our clinic for almost 5 years (at that time, he was only seeing his gastroenterologist) and returned for a follow-up visit only in the year 2018, almost 14 years after the LT. The total regression of xanthomas was evident at the last visit. At that point, he was almost 30 years old, employed, and had a child who was scheduled for a later visit at our lipid clinic and was confirmed to be heterozygote for the same mutation. His work was mostly sedentary, and he was not very physically active. In addition, he started smoking. He was still taking tacrolimus, but no antilipemic treatment was prescribed to him after the LT. His TC and LDL-C levels were 7.2 mmol/L (278 mg/dl) and 5.3 mmol/L (205 mg/dl), respectively. In addition, an elevated ApoB level was present (1.48 g/L), while his Lp(a) level was normal (145 mg/L). Furthermore, hsCRP was 2.11 mg/L. His carotid IMT level had increased to 0.90 mm on both carotid arteries (abnormal result for his age) (15). He was normotensive (97/67 mmHg), and his BMI was 18 kg/m^2^. Liver enzyme levels were in the normal range. The HbA1c level was 4.8%, and the fasting insulin level was 4.3 mE/L. The glomerular filtration rate was above 90 ml/min. At the last visit to our lipid clinic, the patient was recommended to start statin treatment (rosuvastatin, 10 mg) and was transitioned to the adult lipid clinic, where his first visit was scheduled in early 2019. At that visit, his TC and LDL-C levels were 7.36 mmol/L (284.6 mg/dl) and 5.9 mmol/L (228.15 mg/dl), respectively.

## Discussion

HoFH frequently leads to early cardiovascular death if not adequately treated. However, the standard therapy with medications and LDL-apheresis at least in more severe cases often fail to address difficult clinical situation satisfactorily (16–18). The management of patients with HoFH represents a medical challenge despite the approval of new lipid-lowering agents (i.e., mipomersen, lomitapide, PCSK9 inhibitors) (19). An individualized approach in the management of the disease is of great importance (20). Diagnosis of FH and its early treatment is recommended in all guidelines (10, 21–23).

Lipid-lowering drug therapy is recommended for the treatment of HoFH in all age groups (24). LDL-apheresis has been used and progressively became a mainstay in the management of HoFH. In line with current guidelines, treatment should be started as soon as possible, ideally by age 5 and not later than 8 years. However, this and the frequency of treatment represent a compromise between access to centers, the severity of the disease, and the patient's choice and/or compliance (21, 25, 26). Currently, LDL-apheresis is recommended at weekly or biweekly intervals with concurrent administration of maximal doses of lipid-lowering agents in exceptional circumstances such as pregnancy; more frequent treatment without statins may be considered (4, 27, 28).

Despite the lack of randomized studies, there is clinical evidence that long-term lipoprotein apheresis can contribute to plaque regression and/or stabilization, slow coronary atherosclerosis progression, and improved prognosis (29). Thompson et al. have concluded in a 50 year follow-up study that improved treatment and treatment to lower TC levels have a better prognosis (30, 31). Combining apheresis with additional drug therapy to slow down the rapid rebound of LDL-C, which follows each procedure, and to keep the LDL-C level as low as possible for as long as possible is also essential (32, 33). A more recent study has shown that achieving a mean LDL-C level of 4.2 mmol/L (162.4 mg/dl) by weekly apheresis plus statin/ezetimibe therapy failed to prevent the progression of the aortic, coronary, and carotid disease (28, 34). The most recent statement on target levels for both HoFH and HeFH, which advocates lowering LDL-C to <3.5 mmol/L (135 mg/dl) in children and to <2.5 mmol/L (97 mg/dl) in adults, or even <1.8 mmol/L (70 mg/dl) in those at the highest risk can seldom be achieved in homozygotes with existing apheresis/drug therapy regimens (2, 10, 24, 35).

When our patient was 5 years old, biweekly LDL-apheresis first without and then with statins was used. Despite the early treatment initiation, he has developed valvular aortic stenosis. One week after LDL-apheresis, LDL-C levels were up to 10 mmol/L (386.7 mg/dl).

Therefore, LT was considered as a therapeutic option (19, 36), as it was also considered in 23 case reports and case series (19, 36–57), with a total of 90 patients with FH who have undergone LT. Data are presented in detail in Supplement Table 1. Of the 90 patients found, 47 were female. Most of them had HoFH (77.8%) (genetic or clinical diagnosis). Eight patients were just diagnosed with FH. Most of the patients had some kind of CVD before the LT, some having coronary stenting or dilatation (22 patients) or heart valve replacement (4 patients) before LT. Four patients also received a heart transplant in the same surgical procedure, and one also received a kidney transplant at the same time as LT. Although a successful therapeutic strategy (41, 42, 46, 47), there are obvious disadvantages, including the risk of post-transplantation surgical complications and mortality, the risk of acute or chronic organ rejection, and the need for life-long treatment with immunosuppressive therapy (58, 59). In most case studies (63.3%), the cardiovascular disease burden after LT was comparable to the one before LT. In 24.4%, however, the progression of CVD was observed, and in 12.62%, regression was observed (19, 36–57). The latter was also the case in our patient. Cardiovascular complications and disease progression were observed in 11 patients. Of them, six died because of heart failure after LT (19, 36, 38–40, 42, 45, 52). Another two patients died because of septicemia (19, 36). Interestingly, there were no deaths in the five patients with combined heart and liver transplantation (41, 43, 51, 53, 55). One patient lived for 20 years with a combined heart–liver transplant with an absence of CVD (41).

Corticosteroids, cyclosporine, tacrolimus, and sirolimus, the main treatments after transplantation, are all associated with elevated cholesterol levels (60). Cyclosporine interferes with the binding of LDL-C to LDL receptors and also interferes with bile acid synthesis acting on the enzyme 26 hydroxylases. Tacrolimus has similar but lesser effects on lipid metabolism, then cyclosporine (37). In addition, metabolic syndrome can develop after transplantation (19). In our patient, this was not the case. After successful LT, the cardiovascular complications regressed, endothelial dysfunction was not detected, and IMT was reversed.

**Figure 2:**
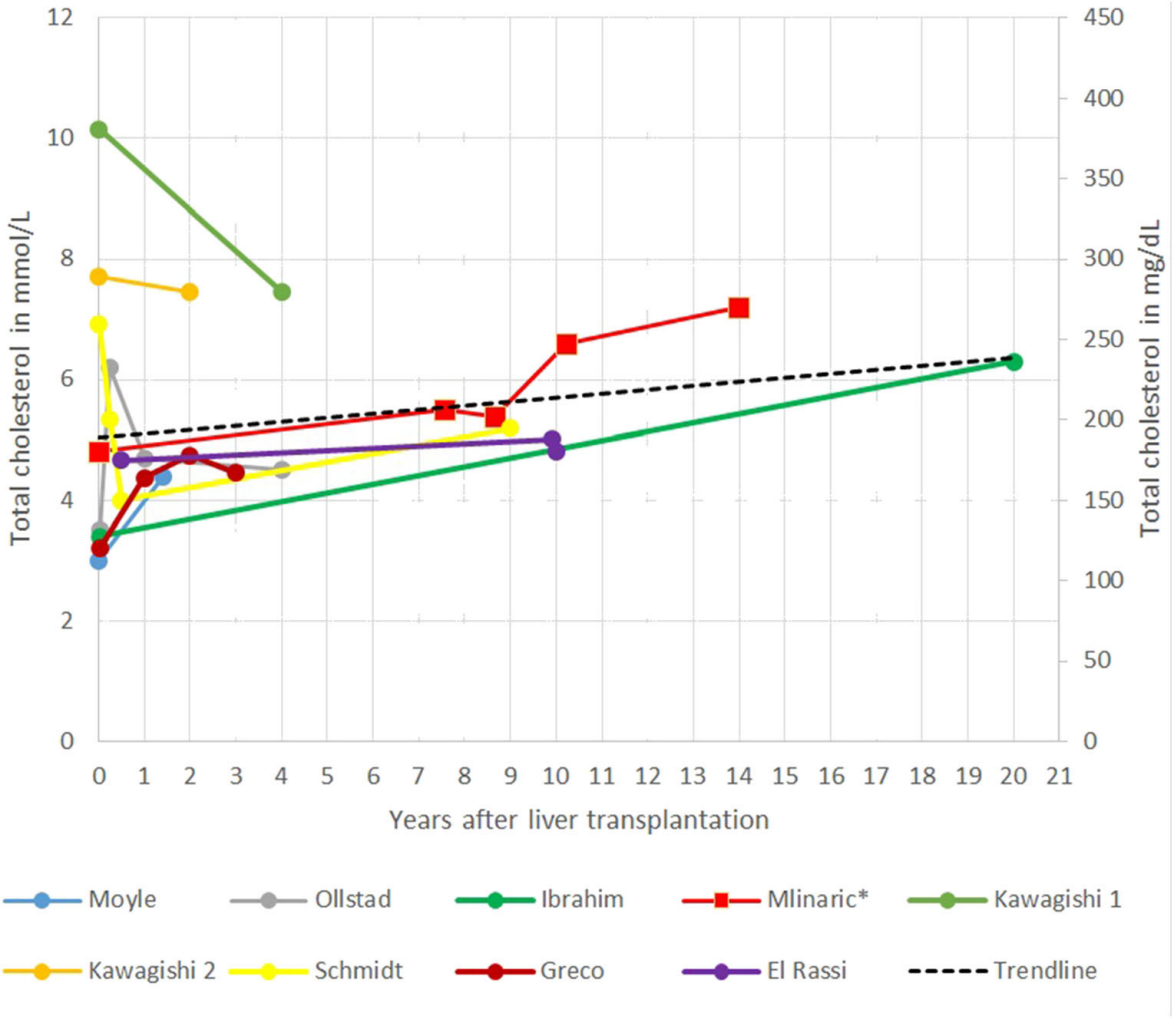
Representation of total cholesterol (TC) after liver transplantation (LT) in different case reports.

In the gathered case reports and our case report interestingly in the short term, a decrease in TC levels after LT was seen, reaching the lowest level in the first years after LT. The main limitation of systematic review of the case reports is that most of the follow-up data were short termed; only a few case reports have followed patients for 10 or more years after LT. The longest report is 20 years (21, 22). Afterward, TC seems to increase steadily, in some patients, even despite taking cholesterol-lowering drugs (Figure 2) (51). Ibrahim et al. (41) report of an increase in TC levels from 3.4 mmol/L (132 mg/dl) to 6.3 mmol/L (244 mg/dl) 20 years after LT. An unhealthy lifestyle (excessive dietary intake of cholesterol and saturated fats), diabetes, obesity, proteinuria, age, genetic predisposition, and medications are known risk factors for elevated cholesterol levels in patients after LT for different defects (37). Some of them are also present in our patient.

Out of the 16 guidelines (2, 4, 21, 22, 27, 61–71) proposed by professional societies and one by Raal et al. (10), 6 of the 17 did not take LT as a treatment option in consideration at all (2, 22, 64, 68, 69). In the guidelines that do mention LT, the majority suggests that it as an exceptional therapeutic option or as the last resort option when all the other treatment options are inadequate or not tolerated (4, 10, 22, 61–63, 65). The International FH foundation states that the LT decision should be in partnership with the patient and/or their relatives (4). In the article by Raal et al. (10), the LT is an acceptable option only for the treatment of HoFH patients that are unresponsive to conventional lipid-lowering therapy and possibly before the onset of significant CVD. However, if the CVD is discovered in preoperative cardiac investigations, the FH Australasia Network consensus group suggests that coronary artery bypass surgery and/or aortic valve replacement should also be considered (62). The same is proposed by the International FH group (4), also suggesting that a combined heart and LT should be considered according to the clinical context. EAS recommends that we should also have in mind the disadvantages of LT (i.e., the need for life-long immunosuppressive therapy, the paucity of donors, and possible surgical complications) (21).

LT is, therefore, especially indicated for patients with HoFH that do not otherwise respond to maximal medical therapy (4, 61–63, 65), but it is not a feasible option for all HoFH patients. However, LT has substantiated the development of other novel therapeutic approaches for patients with severe FH (e.g., liver-direct gene delivery, stem cell transplantation) (67).

The decision should be made with the patient and his relatives in an appropriate setting, and all the benefits or potential harms of the transplantation and of declining LT should be explained (22).

## Conclusions

LT is a feasible therapeutic option, especially in patients with HoFH with progressive atherosclerotic disease that cannot be sufficiently controlled by medications and/or LDL-apheresis. In our HoFH patient, the long-term outcome after the LT was considered highly favorable, reversing the already severely progressed atherosclerosis before the procedure, despite the relatively early detection and intensive treatment with LDL-apheresis.

Replacing the liver where most of the LDL-C metabolism occurs represents a way of somatic gene therapy for HoFH, a frequently fatal inborn metabolic disorder. On the other hand, LT exposes the patient to considerable other clinical risks and burdens associated with the transplantation (e.g., the need for life-long immunosuppressive therapy, the paucity of donors, and possible surgical complications) (21). According to our experience and from the case reports, the LDL-C levels are slowly increasing years after the LT due to an unhealthy lifestyle, tacrolimus, or other therapy (37) and maybe because of LT organ regeneration with cells stemming from the patient. CVD and death of myocardial infarction are also reported in HoFH patients after LT (19, 39). LT might not represent the definite treatment of HoFH. Thus, after LT, the patients need to be further regularly followed at the lipid clinic (and also by the hepatologist), but at least in our patient, the LT vastly improved his quality of life (10, 19). The data indicate that early transplantation may be favorable and that actively preventing septicemia might be improving outcomes.

Finally, early detection programs and the clinical availability of novel therapeutic strategies are urgently needed to address the needs of all HoFH patients. Developing specific guidelines and international patient registries on LT for HoFH might be beneficial, following the example of the research group, who have made the International Registry on Lipoprotein Apheresis in Children with Homozygous Familial Hypercholesterolemia (72).

## Data Availability Statement

All datasets generated for this study are included in the article/Supplementary Material.

## Ethics Statement

Ethical review and approval was not required for the study on human participants in accordance with the local legislation and institutional requirements. The patients/participants provided their written informed consent to participate in this study. Written informed consent was obtained from the individual(s) for the publication of any potentially identifiable images or data included in this article.

## Author Contributions

MM collected the data and prepared the manuscript. NB and UG followed and treated the patient, collected the data, and prepared the first draft of the case report. VD made the first suspicion of the disease and referred him early for further evaluation and treatment and reviewed the manuscript. AS performed follow-up tests and reviewed the manuscript. MC, UG, and TB supervised the work and helped writing the manuscript. All authors approved the final manuscript as submitted and agreed to be accountable for all aspects of the work.

## Conflict of Interest

The authors declare that the research was conducted in the absence of any commercial or financial relationships that could be construed as a potential conflict of interest.

## Abbreviations

FH: familial hypercholesterolemia
HoFH: homozygous familial hypercholesterolemia
HeFH: heterozygous familial hypercholesterolemia
LDL-C: LDL-cholesterol
HDL-C: HDL-cholesterol
TC: total cholesterol
Tgc: triglycerides
LT: liver transplantation
CVD: cardiovascular disease
RHI: reactive hyperemia index
PWV: pulse wave velocity
IMT: intima-media thickness
BMI: body mass index.
