# Supplementary material for: Case Report: Liver Transplantation in Homozygous Familial Hypercholesterolemia (HoFH)—Long-Term Follow-Up of a Patient and Literature Review
Source: Front Pediatr. 2020 Oct 9;8:567895. doi: 10.3389/fped.2020.567895 (PMC7581712; doi:10.3389/fped.2020.567895)
Supplement: Supplementary file 1 [file Data_Sheet_1.docx]

Supplement Table 1: Summary of the 23 identified previous reports of liver transplantation (LT) in familial hypercholesterolemia.

| Report | Number of LT | Number of HoFH patients | Number of males (females) | Highest recorded TC (LDL-C) before LT (mmol/L (mg/dL)) (presented as medians) | Age at LT in years | Donor type | Other organs transplanted with liver | Cardiovascular complications after LT | TC (LDL-C) after LT (mmol/L (mg/dL)) (presented as medians) | Length of follow up (months) | Number of acute rejections | Number of deaths at follow up | Cause and time of death after LT |
| --- | --- | --- | --- | --- | --- | --- | --- | --- | --- | --- | --- | --- | --- |
| Akdur et al (16) | 4 | NS | (4) | NS | 11-19 | 3 LD 1DD | 0 | None | Normal | 10-99 | 1 | 0 | 0 |
| Alim et al (17) | 8 | 8* | 5 (3) | 15.8 (611) (14.8 (574)) | 10 (M) | 5 LD; 3DD | 0 | None in 6, progressions in 2 (sudden cardiac arrest) | 4.6 (170) (3.0 (117)) | M=44 | 0 | 2 | 2 cardiac (1st 2 months, 2nd 18 months) |
| Greco et al (18) | 1 | 1 | 1 | 26.4 (1019) (24.5 (946)) | 9 | NS | 0 | Progression (aortic valve replacement) | 4.6 (167) (2.0 (78)) | 36 | 1 | 0 | 0 |
| Martinez et al (19) | 8 | 5 | 3  (5) | 22.8 (882) (21.7 (820)) | 2-17 | NS | in 1 liver + kidney | 2* had progression in their aortic stenosis; CAD: mild progression in 1, CAD regression in 2 | 4.3 (166) (2.7 (104)) | 24-72 | 2 | 1 | Septic in 1 |
| Mansoorian et al (20) | 36 | 30* | 20  (16) | (24= >12.9 (>500)  3= <10.3 (<400)  9 in between) | 2.5 -28 | 5 LD, 31 DD | 0 | NS (2 had a cardiac arrest) | (3=>5.2 (>200)  14= <3.4 (<130) 16 inbet-ween) | 6-72 | NS | 3 | Cardiac in 2, septic in 1 |
| El-Rassi et al (21) | 1 | 1 | (1) | 29.7 (1150) (27.4 (1060)) | 5 | NS | 0 | Progression (Aortic supravalvular stenosis; severe stenosis of the left and right coronary) | 4.5 (175) (2.6 (100)) | 120 | 0 | 1 | Cardiac and septic in 1 |
| Ibrahim et al (22) | 1 | 1* | (1) | 23.0 (889) (21.0  (812)) | 33 | NS | +heart | None (Absence of CVD) | 6.3 (244) (4.3 (166)) | 240 | 1 | 0 |  |
| Palacio et al (23) | 2 | 1* | 1 | >25.9 (>1000) | / | DD | 0 | Regression(minimal improvement in luminal caliber) | 3.9 (152) (1.9 (75)) | 36 | 1 | 0 | 0 |
|  |  | 1* | (1) | 23.3 (900) | 11 | NS | 0 | None | 5.0 (192) (3.6 (141)) | 6 | 0 | 0 | 0 |
| Alkofer et al (24) | 1 | 0 | 1 (0) | 8.0  (309) | 40 | NS | +heart | None | suboptimal | 36 | 1 | 0 | 0 |
| Khalifeh et al (25) | 1 | 1 | (1) | 26  (1005) | 4 | LD | 0 | NS | 6 (232) | 12 | 1 | 0 | 0 |
| Shrotri et al (26) | 4 | NS | 4 (0) | NS | 10-17 | NS | 0 | NS | normal TC | 12- 108 | 1 | 1 | Cardiac |
| Küçükkartallar et al (27) | 3 | 3 | 1 (2) | 25.9-28.4)  (1000-1100) | 9-14 | 3LD | 0 | None | 3.8-4.7  (146-180) | 8-10 | 0 | 0 | 0 |
| Maiorana et al (28) | 1 | 1 | (1) | (21.3 (824)) | 7.8 | LD | 0 | NS | (3.3±1.3 (128±50)) | 14 | 0 | 0 | 0 |
| Kawagishi et al (29) | 2 | 1 | 1 | 23.3 (898) (19.6 (756)) | 8 | LD | 0 | None | 7.2 (280) | 48 | 0 | 0 | 0 |
|  |  | 1 | (1) | 22.2 (857) (17.8 (689)) | 2 | LD | 0 | None | 7.2 (280) | 24 | 0 | 0 | 0 |
| Schmidt et al (30) | 1 | 1 | (1) | 27.2 (1050) | 16 | NS | 0 | Regression of CAD | 5.0 (195) (3.5 (136)) | 108 | 0 | 0 | 0 |
| Lopez- Santa mariaet al (31) | 2 | 1* | 1 (0) | 29.0 (1120) | 18 | NS | 0 | NS | 3.3 (129) (2.5 (98)) | 13 | 1 | 0 | 0 |
|  |  | 1* | (1) | 23.5 (910) | 16 | NS | 0 | NS | 5.8 (225) (4.8 (186)) | 7 | 0 | 0 | 0 |
| Offstad et al (32) | 1 | 1 | (1) | 25-30 (967-1160) | 46 | NS | +heart | None | 4.5 (174) (2.0 (77)) | 48 | 1 | 0 | 0 |
| Popescu et al (33) | 1 | 1 | (1) | 20.7 (800) | 21 | LD | 0 | None in CAD, improvement in general | 3.0 (116) | 24 | 0 | 0 | 0 |
| Van  Heyningen et al (34) | 1 | 1* | (1) | 187 | 17 | NS | +heart | Progression (coronary stenting) | 4.4 (170) | NS | 1 | 0 | 0 |
| Moyle et al (35) | 1 | 1* | (1) | 28.3 (1094) | 3.5 | NS | 0 | NS | 4.4  (170) | 17 | 0 | 0 | 0 |
| Ahualli et al (36) | 1 | 0 | 1 | >20.7. (>800) | 22 | NS | +heart | NS (mild acute rejection of heart transplant) | <5.2 (<200) | 12 | 1 | 0 | 0 |
| Kirnap et al. (37) | 8 | 8^a^ | 3(5) | ~ 18.1 (698) (~14.4. (554)) | 5-25 | 4 DD, 4 LD | 0 | No CVD progression | ~4.4 (169) (~2.6 (102)) | 24-144 | NS | 0 |  |
| Cephus et al. (38) | 1 | 1 | 1(0) | 26.7 (1043) (25.7 (992)) | 6 | NS | 0 | Stable severe coronary artery disease | 3.3 (126) (1.7 (66)) | 42 | 0 | 0 | 0 |
| ^a^ not genetically confirmed, ^b^out of 4 patients, * Diagnosis according to clinical criteria  Legend: M= median, HoFH=homozygous familial hypercholesterolemia, LDL-C= LDL cholesterol, TC= total cholesterol, CVD =cardiovascular disease, MMF = mycophenolate mofetil, NS= not exactly stated in the articles, LD=living donor, DD=deceased donor | | | | | | | | | | | | | |
